# Development of an Untargeted Metabolomics Strategy to Study the Metabolic Rewiring of Dendritic Cells upon Lipopolysaccharide Activation

**DOI:** 10.3390/metabo13030311

**Published:** 2023-02-21

**Authors:** Jessica Michieletto, Aurélie Delvaux, Emeline Chu-Van, Christophe Junot, François Fenaille, Florence A. Castelli

**Affiliations:** CEA, INRAE, Département Médicaments et Technologies pour la Santé (DMTS), MetaboHUB, Université Paris-Saclay, 91191 Gif-sur-Yvette, France

**Keywords:** sample preparation, liquid chromatography, high-resolution mass spectrometry, dendritic cells, immunometabolomics, metabolomics, adherent cells

## Abstract

Dendritic cells (DCs) are essential immune cells for defense against external pathogens. Upon activation, DCs undergo profound metabolic alterations whose precise nature remains poorly studied at a large scale and is thus far from being fully understood. The goal of the present work was to develop a reliable and accurate untargeted metabolomics workflow to get a deeper insight into the metabolism of DCs when exposed to an infectious agent (lipopolysaccharide, LPS, was used to mimic bacterial infection). As DCs transition rapidly from a non-adherent to an adherent state upon LPS exposure, one of the leading analytical challenges was to implement a single protocol suitable for getting comparable metabolomic snapshots of those two cellular states. Thus, a thoroughly optimized and robust sample preparation method consisting of a one-pot solvent-assisted method for the simultaneous cell lysis/metabolism quenching and metabolite extraction was first implemented to measure intracellular DC metabolites in an unbiased manner. We also placed special emphasis on metabolome coverage and annotation by using a combination of hydrophilic interaction liquid chromatography and reverse phase columns coupled to high-resolution mass spectrometry in conjunction with an in-house developed spectral database to identify metabolites at a high confidence level. Overall, we were able to characterize up to 171 unique meaningful metabolites in DCs. We then preliminarily compared the metabolic profiles of DCs derived from monocytes of 12 healthy donors upon in vitro LPS activation in a time-course experiment. Interestingly, the resulting data revealed differential and time-dependent activation of some particular metabolic pathways, the most impacted being nucleotides, nucleotide sugars, polyamines pathways, the TCA cycle, and to a lesser extent, the arginine pathway.

## 1. Introduction

Untargeted metabolomics aims to measure the largest number of metabolites present in a biological sample, i.e., the metabolome. Metabolomics has been recognized as providing the best reflection of the response of a biological system to any alteration. Liquid chromatography coupled to high-resolution mass spectrometry (LC-HRMS) represents the most widely used technique for untargeted metabolomics [1,2], thus allowing the efficient detection of a large set of structurally diverse metabolites spanning a large range of concentrations in different biological samples. 

In vitro cell models have many advantages in terms of experimental control, availability and biological interpretation of data over analyses of biofluids (plasma or urine). However, they also have particular weaknesses, as special attention must be paid to the sample preparation to avoid any artefactual modifications of the metabolism. Thus, the harvesting, metabolism quenching, and metabolite extraction steps must be specifically adapted to obtain unbiased snapshots of the intracellular metabolome. For this purpose, some metabolomics protocols have been described to study the intracellular metabolite profiles of primary cells, cell lines or cancer cells in culture [3,4,5]. While those protocols have been developed for adherent or non-adherent cells from mammalian sources [3,6], none are yet reported for cells transitioning from non-adherent to adherent states under certain conditions, such as activation of antigen-presenting cells (APCs) like dendritic cells (DCs) or macrophages, when exposed to Toll-Like Receptors (TLRs)-agonists [7,8,9]. Recognition by TLRs of Pathogen Associated Molecular Patterns (PAMPs; bacterial, viral, parasitic, or tumoral patterns) [10,11,12,13,14] allows to detect and signal the presence of a “danger” and leads to the activation of downstream signaling pathways accompanied by dramatic metabolome modifications [15,16]. In TLR-4-activated APCs, some metabolites have been described as critical in the immune response, like lactate promoting external signaling for T cell infiltration [17], succinate leading to further HIF-1α activation and production of the pro-inflammatory cytokine IL-1β [18,19], citrate involved in the production of prostaglandins, nitric oxide [20] while also generating itaconate exhibiting antimicrobial effects [16,21]. More recently, tryptophan/kynurenine derivatives have been largely described as immunoregulators: tryptophan breakdown appears to be associated with the maintenance of tolerance and quinolinic acid acts as a precursor of nicotinamide adenine dinucleotide [22,23]. Immunoregulator metabolites were first observed using targeted analytical approaches (metabolic assays, measurement of Oxygen Consumption Rate (OCR) or Extracellular Acidification Rate (ECAR) in culture cells for assessment of the energy metabolism, etc.), which provided only a limited view of the undergoing metabolic processes. Some studies of the metabolome changes occurring in activated macrophages have been published [13,24,25,26]. However, and to the best of our knowledge, no studies have analyzed large-scale modifications in the metabolome of TLR-4-activated DCs.

The objective of this study was to develop a reliable and unbiased sample preparation protocol to study the intracellular metabolome of DCs going from non-adherent to an adherent state upon bacterial lipopolysaccharide (LPS) activation. Thus, several techniques for collecting cells and normalizing the resulting extracts were thoroughly examined. The metabolites were analyzed using an untargeted LC-HRMS approach, thus providing the first large-scale metabolic map of DCs. The possibility of monitoring numerous metabolites by such a large-scale untargeted metabolomics workflow evidenced specific metabolites impacted by DC reprogramming. Only a few of them had previously been described as immunomodulatory metabolites or potentially playing key roles in the initiation of the immune response. All other metabolites newly observed as drastically regulated under LPS activation have emerged as interesting research targets to better understand the role of metabolites in establishing the immune response.

## 2. Experimental Design

### 2.1. Chemicals

All analytical grade reference compounds were from Sigma-Aldrich (Saint Quentin Fallavier, France). The standard mixtures used for the external calibration of the MS instrument were from Thermo Fisher Scientific (Courtaboeuf, France): Calmix-positive, for the positive ion mode, consisting of caffeine, L-methionyl-arginyl-phenylalanyl-alanine acetate, and Ultramark 1621, and Calmix negative, for the negative ion mode, consisting of Calmix positive mixture plus sodium dodecyl sulfate and sodium taurocholate. Acetonitrile (ACN) was from Biosolve chemicals (Dieuse, France), formic acid from Fisher Chemical (Illkirch, France), paraformaldehyde (PFA) from Sigma-Aldrich (Saint Quentin Fallavier, France), methanol from VWR Chemicals (Fontenay-sous-Bois, France) and deionized water from Honeywell (Fisher Scientific, Illkirch, France). Internal and external standards (Dimetridazole, AMPA (2-amino-3-(3-hydroxy-5-methyl-isoxazol-4-yl)propanoic acid), MCPA (2-methyl-4-chlorophenoxyacetic acid and Dinoseb, and Alanine ^13^C, 2-Aminoanthracene, Dihydrostreptomycin, Roxithromycin (fragment), ^13^C-glucose, ^15^N-aspartate, ethylmalonic acid, amiloride, prednisone, metformin, atropine sulfate, colchicine and imipramine, respectively) from Sigma-Aldrich (Saint Quentin Fallavier, France).

### 2.2. Generation of Monocyte-Derived Dendritic Cells

PBMCs were isolated from buffy coat of adult anonymous healthy donors who gave informed consent (Etablissement Français du Sang, Rungis, France) by density centrifugation (1000× *g*, 15 min) on Ficoll-Hyperpaque gradients (Sigma Aldrich, Saint Quentin Fallavier, France). The cell ring formed and containing monocytes and lymphocytes can be thus recovered. Three further steps of washing with PBS (Fisher Scientific, Illkirch, France) were performed; the first one at 200× *g*, 10 min (to discard platelets), the second and third ones at 400× *g*, 10 min. Then, PBMCs were counted using TC20 automated cell counter (Biorad, Marnes-la-Coquette, France). Monocytes were then purified from the PBMC fraction using anti-CD14 conjugated magnetic beads following constructor’s protocol (Miltenyi Biotech, Paris, France). Cells were seeded at 7 × 10^6^ cells mL^−1^ in 25 cm^2^ culture flasks with 1000 U mL^−1^ IL-4 and GM-CSF in AIM-V medium. After 5 days of incubation at 37 °C in 5% CO_2_, obtained naive DCs were stimulated with LPS (1 µg mL^−1^) and then incubated for the indicated time (0 h, 2 h, 6 h, 16 h, 24 h, 48 h) at 37 °C in 5% CO_2_. Cells were then resuspended in PBS buffer and counted.

### 2.3. Flow Cytometry Phenotyping of DCs

DCs were prepared as described above. The cells were collected with trypsin and after washing in PBS, they were incubated with Fc blocker (Miltenyi Biotech, Paris, France) for 10 min at 4 °C. Finally, cells were stained for surface markers (CD83-APC, REAfinity and IgG_1_-APC, REAfinity (Miltenyi Biotech, Paris, France); CD86-PE, HLA-DR-PE, HLA-ABC-FITC, CD14-FITC, and IgG_2a_-FITC-PE (Becton Dikinson, Rungis, France), following the manufacturer’s instructions. Cells were fixed with 2% PFA for 15 min at 4 °C and rinsed with buffer (according to the provider protocol) before cytometer analysis. The acquisition of 10,000 events was performed on a Novocyte3000 flow cytometer (ACEA Biosciences, Inc., San Diego, CA, USA). Data were analyzed using the manufacturer software (NovocyteExpress^TM^ V1.1.0.). The gated population was DC cells selected according to their forward scatter and side scatter, and CD marker-specific profile (Figure S1).

### 2.4. Sample Preparation Step

#### 2.4.1. Trypsination and Metabolite Extraction

The totality of the medium containing non-adherent cells was collected in 50 mL Falcon tubes (Dutscher, Issy-les-Moulineaux, France). The adherent cells were further harvested from the flask after 5 min at 37 °C in 5% CO_2_ with 1 mL of TrypLE (1X) solution (Fisher Scientific, Illkirch, France). This mixture was added to the previous collected culture medium and centrifuged at 400× *g* during 10 min. The cells were then counted. Total cells were divided into 1.5 mL tubes (Eppendorf, Montesson, France) at 2 million cells/tube and centrifuged at 600× *g* for 5 min. The supernatants were removed to obtain 2 million of dry DC cell pellets, which were stored at −80 °C until extraction. A volume of 170 μL of ultrapure water was added to the cell pellets after thawing. Samples were vortexed during a few seconds. At this step, 20 µL of each sample were withdrawn for further determining the total protein concentration (Pierce BCA Protein Assay Kit, Thermo Fisher Scientific, Courtaboeuf, France; see below). Then, 350 µL of cold methanol (−20 °C) with internal standards were added to the remaining 150 µL of cell lysate and vortexed. Resulting samples were left on ice for 90 min for protein precipitation. Cell debris were then removed by centrifugation at 20,000× *g* during 30 min and supernatants recovered. The resulting aliquots were then evaporated to dryness under a nitrogen stream at 30 °C using a Turbovap^®^ instrument (Biotage, Uppsala, Sweden) and stored at −80 °C until further analysis. Quality control (QC) samples were obtained by pooling 20 µL of each sample after resuspension with suitable solutions.

#### 2.4.2. Organic Solvent Extraction and Quenching

For non-adherent cells, the supernatant was collected and the flask was rinsed twice with 1 mL of cold PBS and the 2 resulting fractions pooled. The mixture was centrifuged (400× *g*, 10 min) to remove potential dead cells present in the supernatant. Nine hundred microliters of water were added to lyse the cells, followed by 2.1 mL of precooled MeOH (−20 °C) containing internal standard mixture (IS). The samples were then vortexed for 10 s and placed on ice for 90 min. A further centrifugation step at 20,000× *g* for 30 min at 4 °C yielded the protein pellet. The protein pellet is kept for further protein quantification (see below). The 3 mL of supernatant was divided into three 900 µL-aliquots. All resting supernatants were mixed to produce a QC and divided as 900 µL-aliquots. All Eppendorf tubes containing 900 µL of the mixture were evaporated under a nitrogen stream using a TurboVap instrument (Biotage) and then stored at −80 °C until analysis. 

For adherent cells, the supernatant was removed, and the adherent cells were rinsed twice with pre-warmed PBS. One milliliter of MeOH/H_2_O+ IS (70:30, *v*/*v*) precooled at −20 °C was added directly to the flask and the cells were scraped off. This step was repeated three times. The 3 collections were pooled in the same tube and placed on ice for 90 min. Then the same steps as above described for non-adherent cells were applied.

### 2.5. Total Protein Quantification

For the trypsination protocol, BCA quantification (Pierce™ BCA Protein Assay Kit, Thermo Fisher Scientific, Courtaboeuf, France) was conducted on 20 µL of cell lysate. For the samples obtained after extraction with organic solvents, the protein quantification was performed on the pellet obtained after the 90 min methanolic precipitation and resuspension in 200 µL of water with sonication for 10 s at 40% Ampl. In brief, 20 µL from each protein lysate was introduced into a 96-well plate and 200 µL of their working solution was added (50:1 of reagent A and B). In order to build a calibration curve, a dilution series of standard solution was used following the manufacturer’s instructions. After 1 h, absorbance was read at 562 nm using a plate reader (SpectraMax^®^ Plus 384, VWR, Rosny-sous-Bois, France).

### 2.6. LC-HRMS Analyses

Metabolic profiling experiments were performed by optimized protocols routinely used in our laboratory [27,28]. LC-HRMS analyses were performed using a U3000 liquid chromatography system coupled to a Q-Exactive mass spectrometer from Thermo Fisher Scientific (Courtaboeuf, France) fitted with an electrospray source. A combination of 2 complementary Hypersil Golf C18 2.1 × 150 mm, 1.9 μm (Thermo Scientific, ref: 10630204) and SeQuant^®^ ZIC^®^-pHILIC 2.1 × 150 mm, 5 µm (Merck; ref: 1.50460.001) chromatographic columns was used to profile metabolites. Prior to LC-HRMS analysis, dried extracts were resuspended to reach a fixed protein concentration (equivalent to 15 µg/10 µL) using variable volumes of solvents. For metabolite analysis using reversed-phase column coupled with ESI+ detection (designed hereafter as C18(+)), mixture of H_2_O/acetonitrile (95:5, *v*/*v*), containing 0.1% formic acid + external standards is used, while a mixture of 10 mM ammonium carbonate pH 10.5 + external standards/acetonitrile (40:60, *v*/*v*) is dedicated to metabolite analysis on Hydrophilic Interaction Liquid Chromatography columns coupled with ESI-detection (designed hereafter as HILIC(−)). After resuspension with suitable solutions, the samples were centrifuged for a further 10 min to eliminate any remaining cell debris or insoluble material. Diluted QC samples were prepared (1/2, 1/4, and 1/8) and injected in triplicate at the beginning of the sequence for further data pre-processing (see below). Non-diluted QC samples were injected throughout the sequence every 10 samples.

The mass spectrometer was calibrated externally before each analysis in both ESI polarities using the manufacturer’s predefined methods and recommended calibration mixture provided by the manufacturer. The Q-Exactive mass spectrometer was operated with capillary voltage at −3 kV in the negative ionization mode and 5 kV in the positive ionization mode and a capillary temperature set at 280 °C. The sheath gas and the auxiliary gas pressures were set at 60 and 10 arbitrary units of nitrogen gas, respectively. The mass resolution of the analyzer was 50,000 full width at half maximum (FWHM) at *m/z* 200, for singly charged ions. The detection was achieved from *m/z* 85 to 1000 for C18(+) conditions and from *m/z* 50 to 1000 for HILIC(−) conditions.

All raw data were manually inspected using the Qualbrowser module of Xcalibur version 2.1 (Thermo Fisher Scientific, Courtaboeuf, France). Raw files were converted to mzXML format using MSConvert software. Automatic peak detection and integration were performed using the XCMS software package included in the W4M platform [29], which returned a data matrix containing *m/z* and retention time values of features together with the corresponding chromatographic peak areas. Metabolic features of interest were filtered according to three criteria: (i) ratio of chromatographic peak areas obtained for biological to blank samples > 3, (ii) coefficient of variation (CV) of metabolites in the QC samples < 30%, (iii) correlation between QC dilution factors and areas of chromatographic peaks > 70%. Metabolite annotation was realized using our spectral database according to accurately measured masses and chromatographic retention times [28,30]. Our chemical database includes ~1200 metabolites (including mainly endogenous metabolites). Confirmation of metabolite annotation was then achieved by running additional LC–MS/MS experiments performed on a Q-Exactive instrument under higher-energy C-trap dissociation (HCD) conditions. Obtained HCD mass spectra were both manually and automatically matched using MS-DIAL 4.60 software to the spectra included in our in-house spectral database, as described elsewhere [31,32]. For a metabolite to be identified, related ions had to match at least two orthogonal criteria (accurately measured mass, isotopic pattern, MS/MS spectrum, and retention time) to those of an authentic chemical standard analyzed under the same analytical conditions, following the recommendations of the Metabolomics Standards Initiative [33].

### 2.7. Statistical Analyses

Statistical analysis of log-transformed data was conducted using the W4M platform version 3.4.4 [29]. The univariate data analyses included a Wilcoxon signed-rank test, corrected for multiple testing by the Benjamini–Hochberg (BH) procedure, to assess the statistical significance of each compound. Multivariate analyses were used to identify molecular features that discriminate metabolite profiles from two conditions. A non-supervised multivariate analysis (PCA) was used to identify features with discriminative power and to maximize variation between the two groups. Hierarchical classification of the samples and features (using centered and reduced data) was also carried out, and represented in the form of a heatmap (MetaboAnalyst 5.0) [34]. The identified metabolites were imported into the free online web-based platform MetaboAnalyst 5.0 for metabolic pathway enrichment. Thus, the annotated and identified *m/z* features with a Wilcoxon *p*-value < 0.05 (BH-critical value) were imported as their HMDB numbers using the appropriate *Homo sapiens* pathway library. The interpretation of the results was performed after considering data with a *p*-value < 0.05.

## 3. Results and Discussion

To facilitate the study of DCs present in low proportions in circulating human blood (1–2%), in vitro differentiation models have been developed. The first models were proposed in the 1990s and involved the differentiation of isolated human blood monocytes or mouse bone marrow cells into DCs in the presence of GM-CSF and often IL-4 [35]. In this study, CD14+ monocytes were positively selected from PBMCs to obtain an extremely pure monocyte population (>97%). The efficient DC differentiation from purified monocytes when exposed to GM-CSF and IL-4 cocktail can be deduced from the significantly increased cells’ size and granularity (microscopic observation and FSC forward scatter and SSC side scatter FACS analysis), the loss of CD14 marker, and the expression of human DC markers such as HLA and CD86 molecules. Then, DCs were exposed to LPS, a bacteria-derived agent widely used for its ability to specifically bind TLR-4, thereby activating DCs.

**Figure 1:**
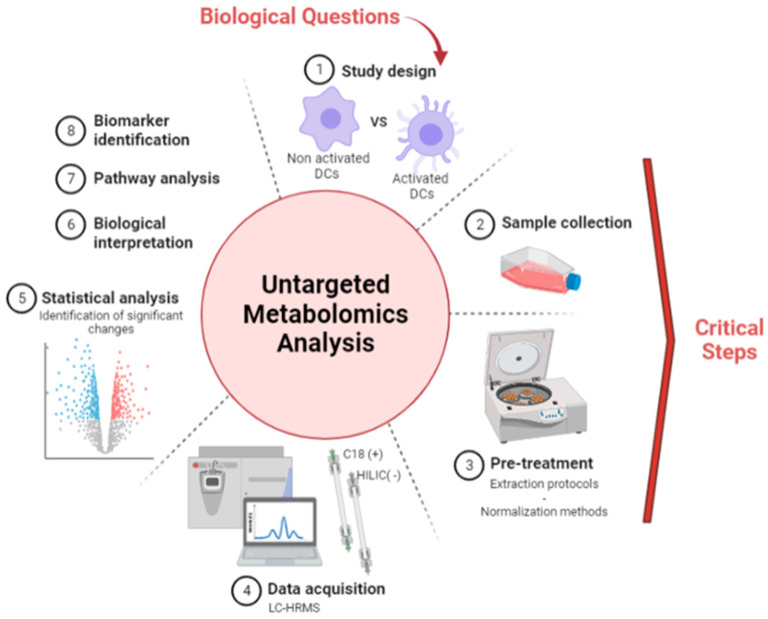
Untargeted metabolomics workflow applied to the study of DCs. Figure made with BioRender.com.

Before applying metabolomic analysis to the study of the DC metabolome, sample collection and normalization steps had to be carefully optimized for the accurate comparison of differentially expressed metabolites in distinct cell extract (Figure 1). A reliable sample collection method is particularly crucial when studying cells transitioning from a non-adherent to an adherent state, as it should strongly influence the detected metabolic fingerprint.

First, we focused on adherent cells by applying a comparative evaluation of 2 distinct experimental protocols: (i) a trypsin-based cell harvesting protocol followed by methanol-assisted cell lysis and metabolite extraction, and (ii) a cold organic solvent method for the simultaneous metabolism quenching, cell lysis, and metabolite extraction directly from cells adhering to the flask. Following protocol optimization, a time-dependent experiment was performed to observe in an unbiased manner the onset of metabolic reprogramming of DCs over 24 h of LPS exposure.

### 3.1. Optimization of Sample Preparation and Normalization Protocols

Four main methods are commonly used for collecting samples from adherent cells, but not all are suitable for the study of the intracellular metabolome. First, using trypsin to detach cells [4,36] is a very common procedure with a high robustness compared to scraping [37], but trypsin seems to have deleterious effects on the intracellular metabolome by altering some metabolite concentrations due to residual metabolic enzyme activities [36,38]. Other “gentle” methods involving ACCUTASE^TM^ or EDTA have also been presented as an alternative to trypsin-based cell harvesting ones. Their main disadvantage is that they require longer incubation times than trypsin depending on the adhesion strength of the cells, leading to potential artifactual metabolite accumulation and/or metabolite leakage [6,39]. Scraping is often used for cell detachment, but does not seem compatible with cellular metabolomics because this mechanical action could damage cell membranes, leading to cell stress and leakage of intracellular metabolites [38,40,41]. The use of cold organic solvents seems ideally suited since such a protocol enables to simultaneously and immediately quench the metabolism, lyse cells, and extract metabolites, thereby avoiding any negative impact on the metabolome [6,38,42].

**Figure 2:**
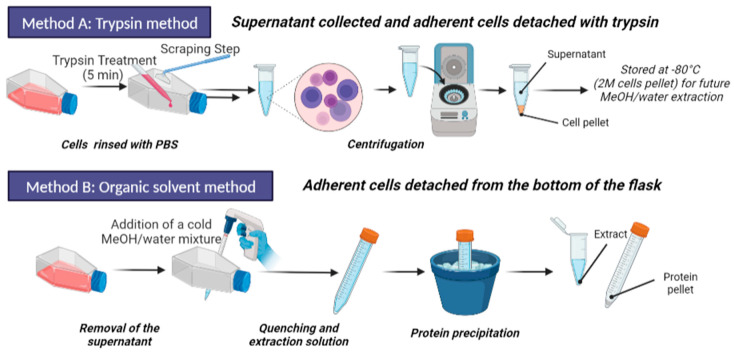
Schematic representation the two evaluated sample preparation methods. Activated (adherent) cells were harvested from the flasks by using trypsin, recovered by scraping, and then lyzed by using MeOH/water mixture (Method A). In parallel, on a culture replicate, adherent cells were directly treated with MeOH/water for simultaneously quenching metabolism, lysing cells, and extracting metabolites (Method B). The cell lysates were analyzed by UHPLC-HRMS for metabolome profiling. Figure made with BioRender.com.

Our objective was to compare the metabolomics profiles obtained using trypsin-based cell detachment followed by cell lysis and metabolite extraction (Method A) and a direct organic solvent protocol involving simultaneous metabolism quenching, cell lysis, and metabolite extraction (Method B) (Figure 2). Normalization of both types of extracts before metabolomics analysis is prerequisite to further successful comparisons of metabolic profiles.

#### 3.1.1. Development of a Sample Normalization Procedure

We first had to set up a sample normalization protocol adapted to both methods A and B to make the comparisons of metabolic profiles accurate (Figure 2). Usually, the normalization of eukaryotic cell samples before mass spectrometry acquisition is based on cell counting. However, it has been shown that this technique might be partially reliable as adherent cells, for example, can aggregate once detached and resuspended, which can impair cell counting [43]. Furthermore, determining the number of cells is not possible with the organic solvents method (method B) because the cells lose their structural integrity once lysed under the action of the solvent [44]. Therefore, we decided to normalize the samples by measuring the total protein concentration using bicinchoninic acid “BCA” protein assay, as it is usually used for tissues or other cellular metabolite extracts before mass spectrometry analyses [45].

The special feature with the samples we wanted to normalize was that proteins to quantify were in the pellet obtained after precipitation with methanol. Indeed, both A and B methods induced protein pelleting following methanol addition, acting as a precipitating agent for proteins. We thus validated the accuracy of the BCA protocol for this particular type of biological material after subsequent centrifugation and resuspension of the pellet of proteins in pure water in both cases. We collected different amounts of cells (5 Million, 4 M, 2.5 M, 2 M, 1.5 M, 1 M, 0.6 M, and 0.5 M after cell counting) that were further precipitated by adding MeOH/H_2_O+ IS (70:30, *v*/*v*). Sonicating the samples following reconstitution with pure water proved mandatory to get homogenous samples and more robust data (Figure S2). We observed that the quantification of total protein amounts from protein pellets well correlated with cell numbers with r^2^ > 0.95 (Figure S3). This excellent correlation between number of cells and total protein concentration in the protein pellet after methanolic precipitation validated our choice to use this total protein concentration as a confidence metric for the sample normalization step.

#### 3.1.2. Comparison of Metabolite Fingerprints Obtained with Trypsin- or Organic Solvents-Based Protocols

DCs obtained from the blood of four healthy donors were cultivated during 5 days and further activated with LPS for 24 h, thereby rendering cells adherent. Methods A and B were performed as described in Figure 2 to get the corresponding metabolite extracts. Sample normalization before LC-HRMS data acquisition was performed using the total protein concentration measured as described in 3.1.1. LC-HRMS metabolomics analyses were performed using two complementary chromatographic conditions involving a C18 with detection in the positive ionization mode [C18(+)] and an HILIC column with detection in the negative ionization mode [HILIC(−)], allowing the analysis of hydrophobic and polar metabolites, respectively (Figure 1). 

Following data processing, we obtained 8517 and 10,470 features in the HILIC(-) and C18(+) analytical conditions, respectively. Of these, 2422 and 2395 features were found to be analytically relevant, i.e., satisfying our 3 filtering criteria: ratio of chromatographic peak areas obtained for biological to blank samples > 3; CV of metabolites in the QC samples < 30%, and correlation between QC dilution factors and areas of chromatographic peaks > 70%. Among these features, we annotated and identified 171 unique metabolites (135 HILIC(-) and 54 C18(+), 18 of which were in common), matching with the accurate RT, *m/z* values, and the MS/MS spectra of pure standards included in our in-house library thus classifying them as “Level 1” metabolites according to the Chemical Analysis Working Group of the Metabolomics Standards Initiative [46] (see Table S1). We noted that this metabolite fingerprint obtained on DCs provided a comparable amount of features and identified metabolites (around 200 annotated metabolites based on *m/z* and RT correspondence) as metabolomics studies conducted in our laboratory in the same analytical conditions on usual matrices for us as plasma [30,47] or tissue [48,49].

**Figure 3:**
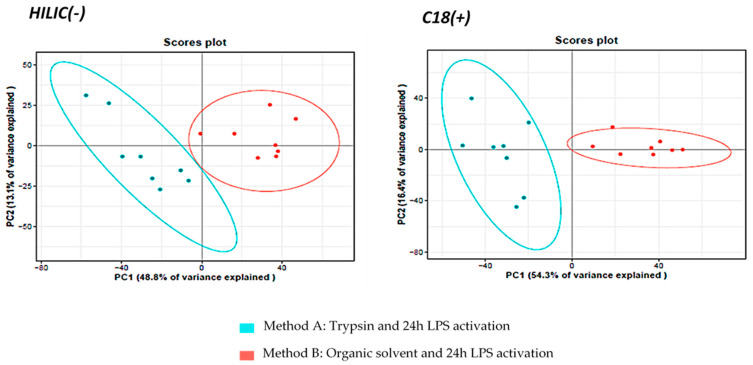
PCA multivariate analysis of the extracted cell samples analyzed under HILIC(−) and C18(+) conditions.

Unsupervised multivariate Principal Component Analysis (PCA) on the metabolite datasets revealed segregation of methods A and B, demonstrating that the sample preparation protocol influenced the metabolomics profile (Figure 3). We checked that the relative abundances of the external standards in both C18(+) and HILIC(−) conditions, were not statistically different (for more than 70% of ES) between the two extraction protocols (Figure S4), in order to rule out the hypothesis that a possible strong matrix effect could have led to the observed clustering. Once this verification conducted, we performed univariate statistical analyses (Wilcoxon–Mann–Whitney test) with BH correction on all the annotated metabolites to characterize further the differences between the two methods A and B. Of the 171 metabolites identified with high confidence and measured simultaneously in DC samples, we discovered a set of 72 metabolites expressed statistically differently in the two methods. Those metabolites are involved in several distinct pathways (Table S2), demonstrating that one of the preparation methods significantly affected the metabolomics profile leading to huge differences in metabolic profiles. We suspected that the bioactive enzyme trypsin was more likely to have an impact on cell metabolism than method B based on the use of simple solvents. Indeed, Teng et al. demonstrated the impact of trypsin on MCF-7 cells [42]. Under the action of trypsin, which cleaves adhesion proteins, cells undergo morphological and physiological changes, as well as metabolic changes, as observed in the present study. We detected many altered pathways like phenylalanine, tyrosine and tryptophan biosynthesis, phenylalanine metabolism, glycine, serine and threonine metabolisms, glutathione metabolism, amino sugar, and nucleotide sugar metabolisms, cysteine, and methionine metabolisms (Table S2). These observations are in good agreement with published data also warning against harvesting with trypsin for cellular metabolomic analyses on adherent mammalian cells [3,6,37]. We also noticed that 80% of the metabolites expressed statistically differently in the two methods are distributed in different metabolic pathways and exhibited decreased concentrations in cell extracts obtained with method A compared to those obtained with method B (Table S2). Such global drop in metabolite concentrations should reflect substantial metabolite leakage following trypsin treatment due to associated membrane damages leading to membrane permeabilization [3]. In addition, we could also observe the accumulation of metabolites statistically and directly associated with the presence of trypsin such as fatty acids like hydroxyhexadecanoic acid, elaidic acid or hydroxyoctadecadienoic acid, as well as adenosine or purines. Rushing et al. also showed an upregulation of fatty acids upon trypsinization which can be related to energy utilization associated with the morphological and physiological modifications of cells specifically observed in the presence of trypsin [50]. Regarding the increase of purines, as these metabolites are involved in the production of NADPH and glutathione, we could hypothesize that trypsin could have an impact on the antioxidant response in cells. Further investigations would be needed to fully understand the exact origin of these changes. Nevertheless, those results clearly demonstrated the presence of strong biases associated with trypsin treatment, making it inadequate for metabolomics studies. In addition, the organic solvent extraction technique maintains satisfactory repeatability CVs of < 20% (n = 4 samples) (Table S3) and is also particularly easy to implement when working on adherent cells. For all these reasons, the protocol involving organic solvents was kept for the following investigations.

### 3.2. Metabolomics Investigation of the Reprogramming Process of DCs upon LPS Exposure

#### 3.2.1. Impact of LPS on DC Phenotype 

To hold their function of sentinels, non-activated DCs monitor their microenvironment (pathogens, antigens, soluble molecules) through phagocytosis, macro-pinocytosis, and receptor-mediated endocytosis [51,52]. The detection of PAMPs by pattern recognition receptors (PRRs) on DC membrane such as TLRs, induces DC activation. We chose PAMPs exposition with LPS, as a well-known model TLR-4 ligand, to induce DC activation. The activation process of DCs is associated with phenotypical and functional changes that allow them to migrate to lymph nodes and induce their ability to activate T lymphocytes. Thus, activation of DCs leads to an increase in the expression of several membrane molecules such as CD83, the co-stimulatory molecules CD80, and CD86, chemokine receptors or the major histocompatibility complex (MHC) molecules that are redistributed to the surface of DCs for subsequent presentation to T cells [53]. We first studied in a time-dependent manner (0 to 48 h) the LPS effect of activation on DCs by monitoring specific membrane markers of activated DCs.

**Figure 4:**
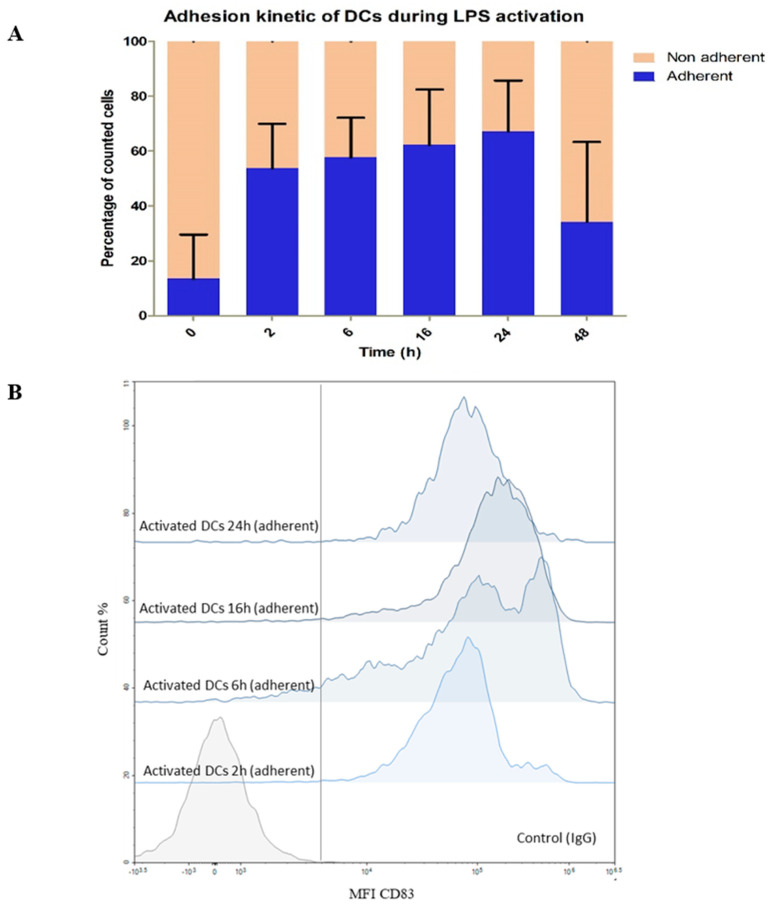
Kinetic experiment over 48 h of LPS−DC activation. 0 h: without LPS; 2, 6, 16, 24, 48 h: exposure time to LPS at 1 µg mL^−1^. (**A**) Bar graphs showing the evolution of the proportion of the DC state (non-adherent to adherent) over time in the culture medium (orange) or attached to the flask (blue). (**B**) Evolution of the surface marker CD83 over time on adherent DC population by flow cytometry analyses. The vertical gray line represents the separation between the negative and positive gates according to the intensity of the IgG anti-mouse control. MFI: Mean Fluorescent Intensity.

Kinetics of LPS activation of DCs was set up with the following time points: 0 h, 2 h, 6 h, 16 h, 24 h, and 48 h. Cellular activation was observed both through using a light microscope through the formation of dendrites, by counting adherent cells in the culture flasks, and with analyses by flow cytometry to detect the expression of CD83 marker (Figure 4). The impact of LPS on DCs was observed as soon as the first 2 h with already up to 45% of adherent DCs, reaching 65% after 24 h of exposure. Then, the population of adherent cells decreases dramatically at 48 h indicating probably cell death as expected because it is well documented that activated DCs have a short lifetime [54] (Figure 4A). This is corroborated by some publications showing the presence of different cellular states upon prolonged LPS exposure (less adhesiveness, appearance of apoptosis phenotype) [54]. We confirmed that adherent cells were indeed activated DCs by measuring the expression of membrane CD83 (only until 24 h), known as being stably expressed by activated DCs. The evolution of the mean fluorescence intensity (MFI) of CD83 was highly positive and increased positively with time for adherent cells (Figure 4B) whereas non- adherent cells were “CD83-low” (data not shown). We also monitored by flow cytometry other membrane molecules (CD14, CD86, and HLA ABC) as DC activation markers and confirmed CD83 data (data not shown). According to these results, we therefore decided to limit the study of metabolomics reprogramming upon LPS activation to 0–24 h, focusing on the time window where the DCs are healthy to avoid an impact of dying cells on their own metabolism or on that of neighboring cells.

#### 3.2.2. Monitoring of DCs and Metabolic Reprogramming upon LPS Stimulation by Untargeted Metabolomics

**Figure 5:**
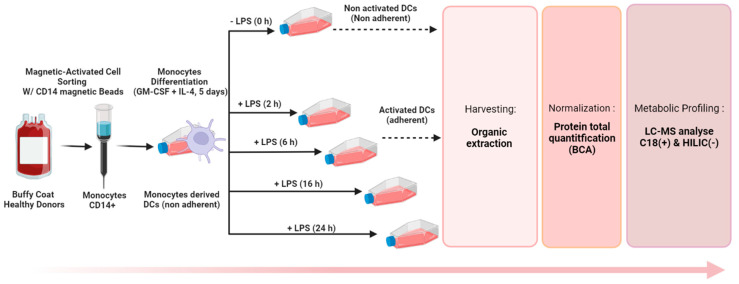
Optimized workflow for the untargeted metabolite profiling of DCs.

We implemented our optimized protocol to monitor the metabolomic reprogramming of DCs upon LPS exposure. Monocytes were isolated from the blood of 12 donors and seeded at 7 million/flask in 5 flasks to undergo differentiation into naive DCs before being exposed to LPS for 5 different times from 0 to 24 h (0, 2 h, 6 h, 16 h, and 24 h). Metabolic extracts collected with method B were analyzed by LC-HRMS after normalization (according to protein content) to study the LPS-metabolic rewiring of DCs (Figure 5). Metabolic profiling experiments were thus performed as described in Section 2.6 on a total of 60 samples: 12 primary cultures of DCs derived from 12 different donors and exposed to LPS during 5 different times. 

**Figure 6:**
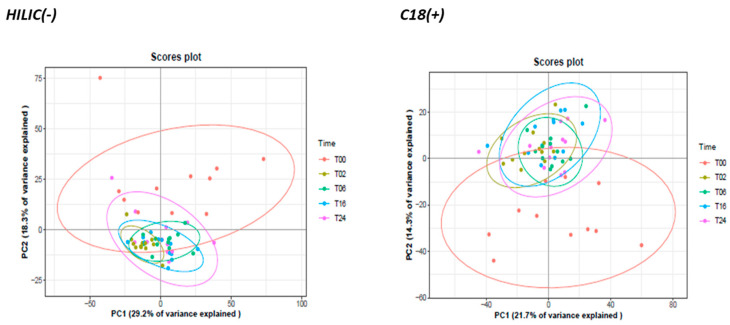
Principal component analysis (PCA) of all detected variables across time in HILIC (−) and C18 (+) analytical conditions. Data were Log10−transformed before PCA.

To get the first rough picture of samples and data distribution of the obtained metabolomics fingerprint, a PCA analysis was performed using all the analytically relevant features obtained under C18(+) and HILIC(−) conditions yielding 1627 and 1359 detected features, respectively. The first two PCA components explained 29% and 18% of the total variance of HILIC(−) data and 22% and 14% for C18(+) data (Figure 6). Both non-supervised PCAs highlighted similar structuration with LPS-activated cells (T2, T6, T16 and T24) clustering apart from naive cells (T0) (Figure 6). Moreover, LPS activation seems to render cell populations more homogenous.

Complementary experiments revealed that incubating cells during 24 h without LPS does not modify their metabolomics profiles (Figure S5). Moreover, all the external standards proved not impacted by the non-adherent/adherent phenotype, thus suggesting the absence of a significant matrix effect and the consistency of the protocol (Table S4–Figure S6). Altogether, these observations demonstrate that differences in metabolic profiles observed in Figure 6 were specific to LPS activation.

**Figure 7:**
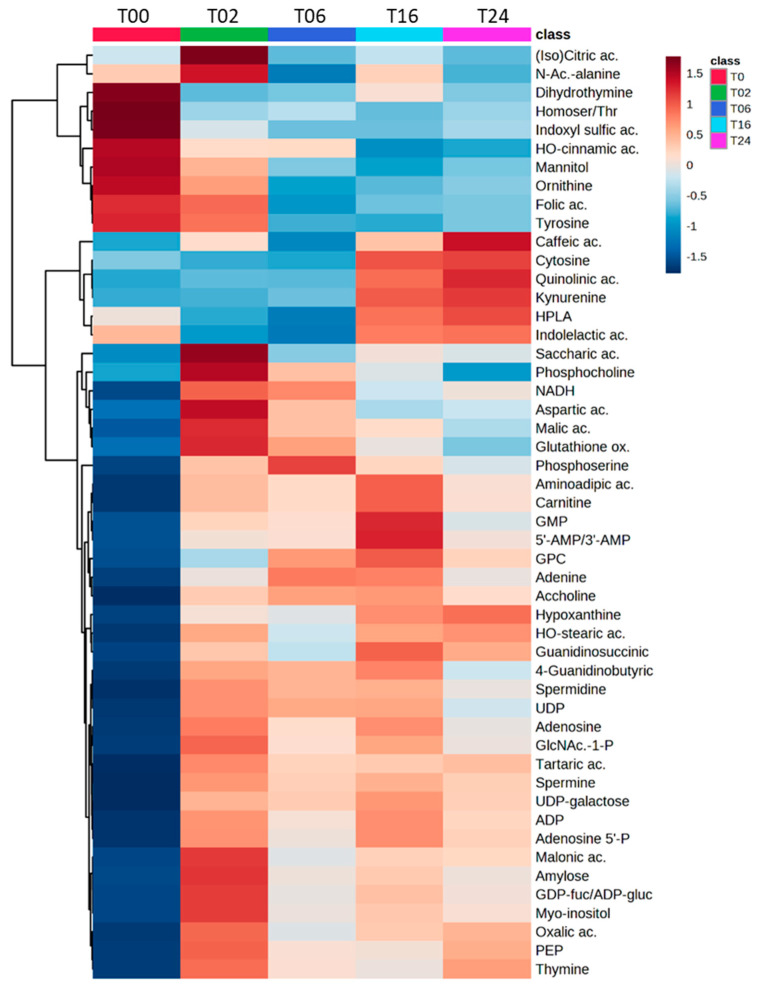
Heatmap showing the modulation of the top 50 impacted metabolites (according to ANOVA *p*-values) upon exposure of DCs to LPS. The heatmap depicted high (red) and low (blue) relative abundances (log-transformed, mean values are represented) of metabolites from HILIC(−) and C18(+) datasets. *Ac.: Acetyl; ac.: acid; ADP: Adenosine diphosphate; ADP*−*gluc: ADP-glucose; GDP: Guanosine diphosphate; GDP*−*fuc: GDP*−*fucose; GlcNAc*−*1*−*P: N*−*acetyl*−*alpha*−*D*−*glucosamine 1*−*phosphate; Gluthanione ox.: Glutathione oxidized; GMP: Guanosine monophosphate; GPC: Glycherophosphocholine; HPLA: Hydroxyphenyllactate; Homoser/Thr: Homoserine/Threonine; NADH: Reduced nicotinamide adenine dinucleotide; P.: Phosphate; PEP: Phosphoenolpyruvate, UDP: Uridine diphosphate*.

In a second step, we generated a cluster analysis heatmap from the annotated part of metabolomics fingerprint (top 50 according to ANOVA *p*-values), and highlighted a temporal evolution of metabolite profiles over the 24 h of LPS exposure, when comparing the different time points of the kinetics (Figure 7 and Figure S7, for a non-averaged view of the groups). A large portion of the annotated metabolites (n = 41, Table S5) accumulated at 2 h and then decreased regularly until 24 h of treatment. Those compounds belong essentially to nucleotides, nucleotide sugars, polyamines pathways, and TCA cycle as well as to a lesser extent to the arginine pathway. Such a large diversity of regulated pathways denoted an important metabolic reprogramming occurring early. In addition, some metabolites belonging to the tryptophan and phenylalanine pathways accumulated at 24 h. 

**Figure 8:**
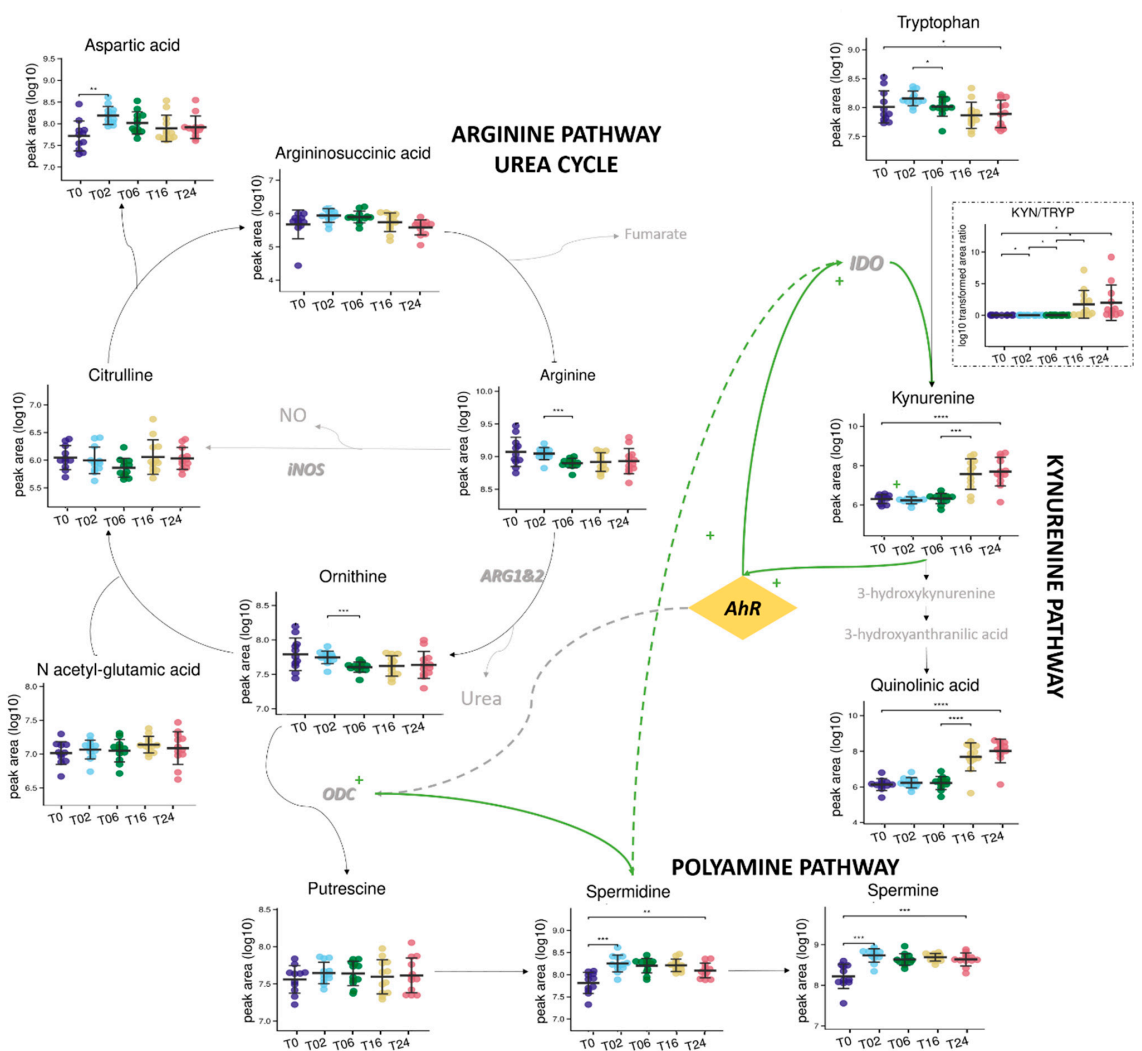
Metabolic coverage of arginine, kynurenine, and polyamine pathways and possible interconnections. Variation in the relative abundance of metabolites (log-transformed, mean values are represented (SD)) from 0 to 24 h of LPS incubation. *: *p*-values < 0.05 **: *p*-values < 0.01; ***: *p*-values < 0.001, and ****: *p*-values < 1 × 10^−4^. Metabolites in gray are not detected, iNOS: nitric oxide synthase; ARG1&2: arginase; ODC: ornithine decarboxylase; IDO: Indoleamine-2,3-dioxygenase; AhR: Aryl Hydrocarbon Receptor.

Forty-nine percent (1469/2986 total features) of the metabolic fingerprint (DC metabolome) were significally affected by the DC reprogramming, and 50 of them were annotated and represented in Figure 7. Interestingly, 12 of these 50 significant and annotated metabolites covered 3 pathways: arginine/urea, polyamine, and kynurenine pathways (Figure 8). Although these metabolites have been studied individually in an hypothesis-driven manner by others in separate publications, this is the first study reporting the use and value of untargeted metabolomics to study those compounds among the 2986 features detected concomitantly in a single analysis and without any a priori regarding their potential biological or biochemical relevance.

More precisely, arginine (Ratio T06/T02: 0.71), proline (Ratio T06/T02:0.64), ornithine (Ratio T06/T02: 0.71) and glutamine (Ratio T06/T02: 0.65) concentrations were decreased, contrary to those of spermidine, a product from the polyamine synthesis part (Figure 7 and Figure 8 and Table S5). Arginine consumption was observed in many pro-inflammatory macrophages [55,56,57] and was due to the action of the enzyme iNOS allowing the production of NO and citrulline. Here, no significant change over time was observed for citrulline (Figure 8), suggesting that arginine degradation is preferentially mediated by Arginase 1 (ARG1) for the production of ornithine which can be metabolized to putrescine by the ornithine decarboxylase 1 (ODC1) enzyme. Subsequent conversion of putrescine to spermidine and spermine gives a set of polyamines characterized by important roles in the control of immune responses and thus described in several inflammatory environments [58,59]. More precisely, spermidine has anti-inflammatory properties and could be secreted to regulate the response of DCs. Indeed, it seemed capable of modulating signaling pathways by acting as a negative regulator influencing the biology of inflammation (by reducing the secretion of pro-inflammatory cytokines for example) [60,61]. As mentioned, during the later stage of the LPS activation (after 6 h), metabolic variations were fewer. Importantly, the concentrations of tryptophan metabolites from the kynurenine pathway were significantly upregulated, which was particularly marked for kynurenine (Ratio T16/T06: 50) and quinolinic acid (Ratio T16/T06: 50) (Figure 8 and Table S5). Recently, Fall et al. [26] detected and quantified similar alterations in pulmonary macrophages, demonstrating the involvement of tryptophan metabolism in the regulation of cell polarization. The concomitant decrease of tryptophan concentration proved also consistent with those observations and highlighting the indoleamine 2,3-dioxygenase (IDO) activation, a well described mediator of inflammation [62]. The kynurenine/tryptophan ratio is a well regarded indicator of IDO1 activity and was found to positively correlate with LPS incubation time. The kynurenine pathway has been described to have a special role during inflammation observed in several diseases by inducing tolerance and increasing production of NAD+ [22,63]. Degradation of tryptophan into kynurenine could or should induce maturation of immature T cells into regulatory T cells or activate resting memory T cells. Those results were similar with published data on LPS-activated macrophages [13,25].

Altogether, our data are consistent with the previously reported central role of AhR in the immune response of DCs, thus linking arginine and tryptophan metabolism [64,65]. AhR activation by kynurenine has been described to increase polyamine production through ODC1 [61], while spermidine can also induce the IDO1 protein thereby triggering tryptophan consumption and downstream production of metabolites from the kynurenine pathway [58]. Previous data also indicated that ARG1 and IDO1 are linked by an entwined pathway mediated by spermidine [58]. Therefore the polyamine-kynurenine-AhR loop is often regarded as an immunoregulatory circuitry in DCs [61].

## 4. Conclusions

The metabolome reflects extra-rapid metabolic reactions, and it is rapidly evolving and defined as extremely dynamic. Acquiring the most accurate metabolic fingerprint of a given experimental state at a given time requires great care in sample preparation. Throughout this work, we have compared different methods of preparing and normalizing DC samples. Optimized sample preparation method using organic solvent for simultaneously quenching/metabolite extraction allowed getting a rapid, reliable, and unbiased snapshot of the metabolism. However, this method leads to the destruction of all the cells in culture, thus precluding its use as a “universal” sample preparation upstream of any other analytical technique requiring the collection of intact cells (e.g., flow cytometry). More broadly, we recommend this protocol for metabolomics analyses of all types of adherent cells (e.g., macrophages, hepatocytes, neurons, fibroblasts, etc.), leaving aside the classical methods used to detach adherent cells such as scraping or the use of bioactive molecules (trypsin, accutase) which would deteriorate membranes and causes metabolite leakage and bias metabolic fingerprints by triggering unwanted metabolism modifications. Our results also showed that LPS drives drastic alteration of the intracellular metabolite content in a time-dependent manner within the 2–24 h window. Interestingly, nucleotides, nucleotide sugars, polyamines, and TCA cycle demonstrated rapid but transient accumulation from 2 h to 16 h. While peaking at 24 h, metabolites belonging to the tryptophan and phenylalanine pathways showed delayed accumulation. These observations are in perfect agreement with the polyamine–kynurenine–AhR immunoregulatory circuitry often described in DCs. These conclusions can be drawn from literature data only by collectively considering several papers targeting the distinct parts of such metabolic circuitry, while the sole present paper allows to get the whole picture in one shot thanks to the implemented untargeted metabolomics approach. Our work also highlights the value of kinetically studying LPS activation to decipher the different phases and components of DC activation. It would be interesting to apply the current methodology to the comparative study of various TLR-specific activators such as LPS or poly I:C and their impact on DC metabolism.

## Data Availability

Raw data are available upon request to the corresponding author. Data are not publicly available due to privacy.

## Supplementary Materials

The following supporting information can be downloaded at: https://www.mdpi.com/article/10.3390/metabo13030311/s1, Figure S1: Flow cytometry dot plots of the gating strategy applied for DC analysis from CD83 marker example. Total DCs were first gated on a forward scatter (FS)/side scatter (SS) plot (a), and then we verified the quality of the acquisition on Time/FSC-A plot. Furthermore, finally, we gated a single cell on the SSC-H/SSC-A plot to eliminate doublets and other aggregated particles. Figure S2: Usefulness of using an ultrasonication step (with a sonication probe) to facilitate cell resuspension. Efficiency and robustness of the approach was evaluated by BCA protein quantification. Each point corresponds to the CV of two technical replicates. Figure S3: Correlation between number of cells precipitated and BCA protein quantification read-out. Each data point is the average of 4 technical replicates, 8 different cell numbers were used (5 Million cells, 4 M, 2.5 M, 2 M, 1.5 M, 1 M, 0.6 M, and 0.5 M after cell counting); Figure S4: Impact of the two sample preparation protocols on the concentrations of external standards during the LC-HRMS analysis in C18(+) (A) and HILIC(−) (B) conditions: (i) use of trypsin to collect the cells followed by a methanolic extraction (trypsin) or (ii) use of the quenching method and simultaneous extraction of the intracellular metabolites by cold methanol (organic solvent); Figure S5: Multivariate supervised statistical analysis was performed on samples from DCs directly after differentiation under GM-CSF and IL-4 during 5 days (T0, blue) and after differentiation +24 h of cell culture in more (Ctrl, red). PLS-DA score plot (R2 = 0.649 and Q2 = 0.23); Data from C18(+) analyses, Log10-transformed; Figure S6: Impact of the cellular state (adherent (0 h), non-adherent (24 h)) on concentrations of external standards during the analysis of the samples analyzed under HILIC(−) and C18(+) conditions; Figure S7: Heatmap obtained from unsupervised analysis of the 12 cell samples collected during 24 h kinetics. Presentation of the 62 most significant metabolites from the t-test/ANOVA analysis (Distance measure: Pearson; Clustering method: Mean). Three large clusters can be distinguished, which correspond to (i) decreased metabolite concentrations within the first two hours, (ii) increased metabolite concentrations after the first 6 h, and at last, increased metabolite concentrations after 2 h; Table S1. List of metabolites annotated in dendritic cell samples; Table S2. List of annotated metabolites and relative chemical classes differing between trypsin/organic solvent methods; Table S3. Coefficient of variation across repeatability experiment for samples under trypsination and under direct solvent extraction (n = 4 samples); Table S4. List of external standards used for DCs’ analysis; Table S5. List of metabolites significantly altered during LPS activation (significant changes over the time). Observation of the evolution over time (comparison Tx+1/Tx).
