# Supplementary material for: Development of an Untargeted Metabolomics Strategy to Study the Metabolic Rewiring of Dendritic Cells upon Lipopolysaccharide Activation
Source: Metabolites. 2023 Feb 21;13(3):311. doi: 10.3390/metabo13030311 (PMC10058937; doi:10.3390/metabo13030311)
Supplement: Supplementary file 1 [file metabolites-13-00311-s001.zip › metabolites-2207280_Supplementary Files Figures_Michieletto.pdf]

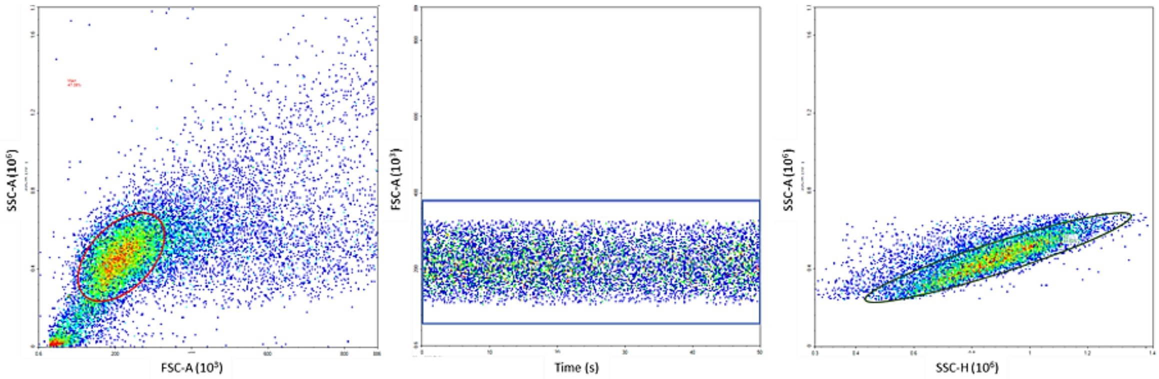

Figure S1: Flow cytometry dotplots of the gating strategy applied for DC analysis from CD83 marker example. Total DCs were first gated on a forward scatter (FS)/side scatter (SS) plot (a), and then we verified the quality of the acquisition on Time/FSC-A plot. Furthermore, finally, we gated a single cell on the SSC-H/SSC-A plot to eliminate doublets and other aggregated particles.

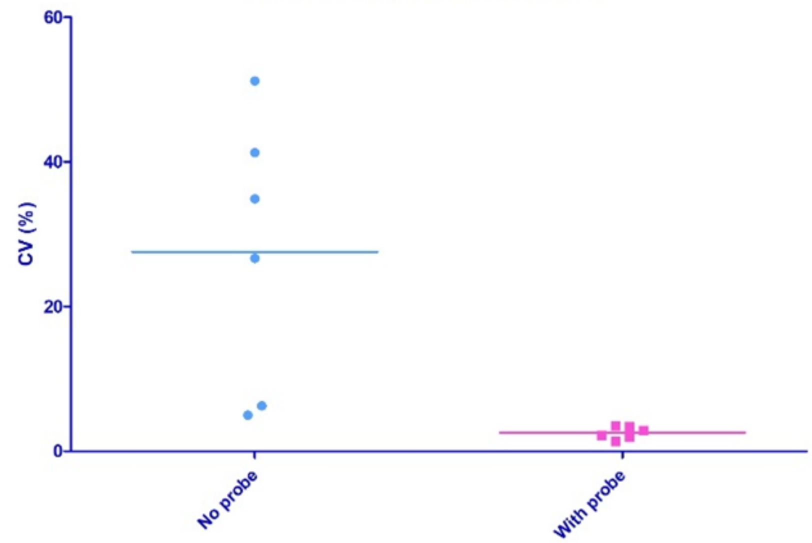

Figure S2: Usefulness of using an ultrasonication step (with a sonication probe) to facilitate cell resuspension. Efficiency and robustness of the approach was evaluated by BCA protein quantification. Each point corresponds to the CV of two technical replicates.

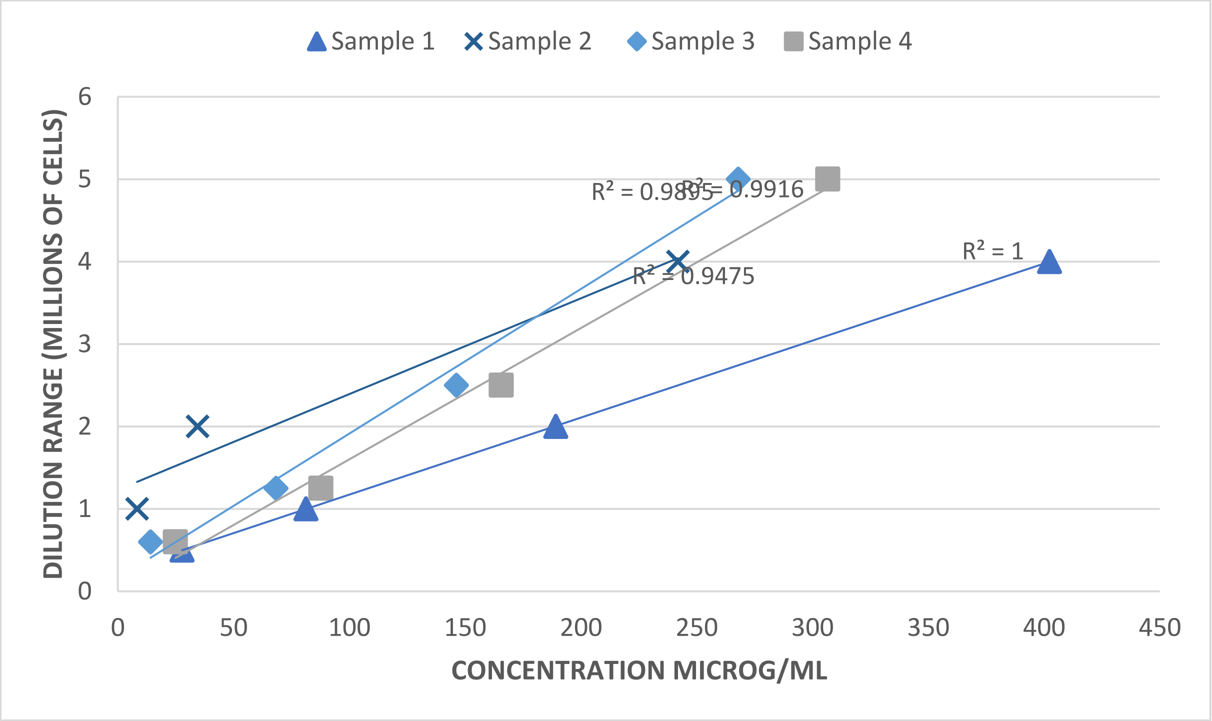

Figure S3: Correlation between number of cells precipitated and BCA protein quantification read out. Each data point is the average of 4 technical replicates, 8 different cell numbers were used. (5 Million Cells, 4 M, 2.5 M, 2 M, 1.5 M, 1 M, 0.6 M and 0.5 M after cell counting).

(A)

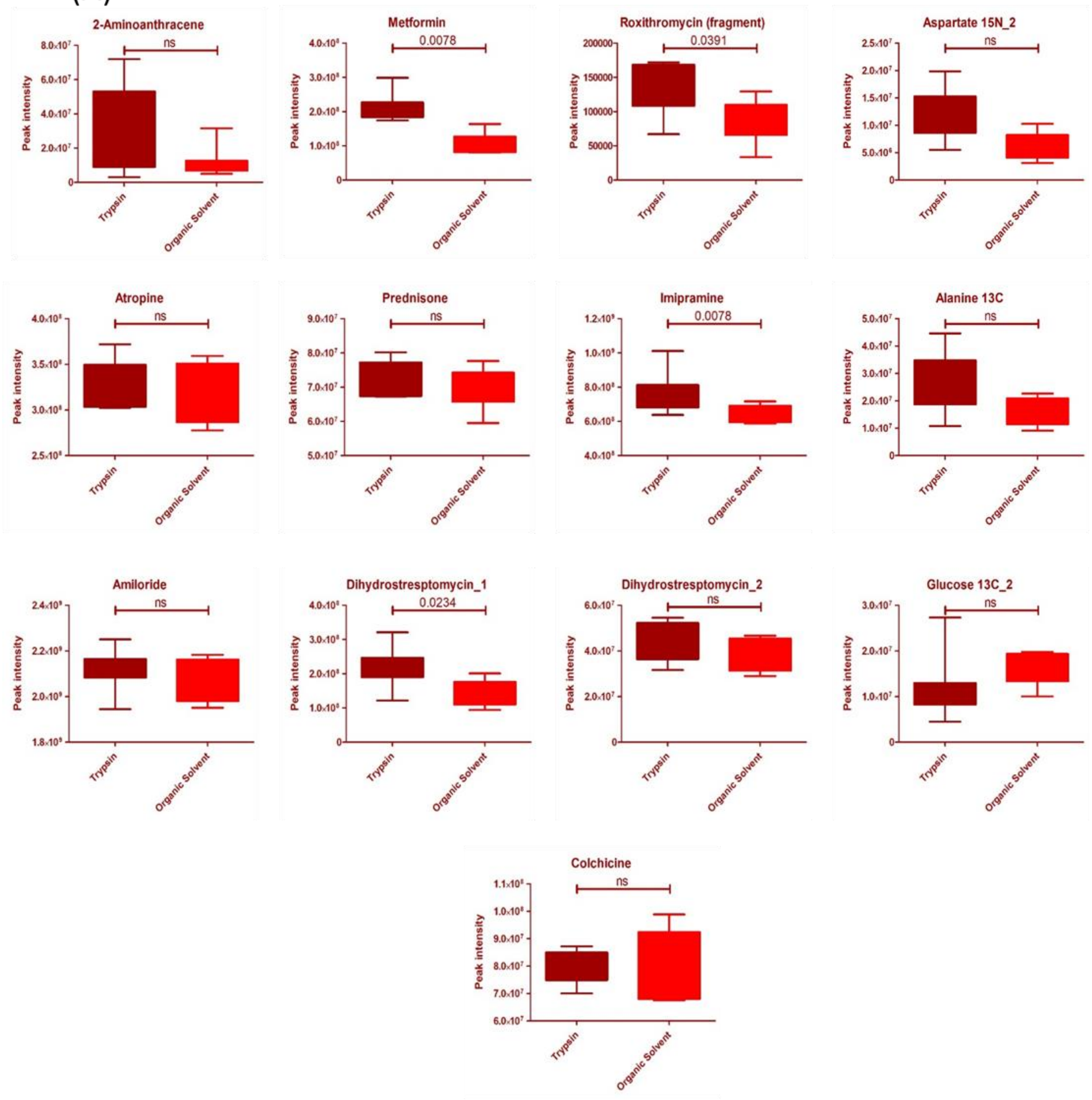
2-Aminoanthracene
Metformin
Roxithromycin (fragment)
Aspartate 15N_2
Atropine
Prednisone
Imipramine
Alanine 13C
Amiloride
Dihydrostresptomycin_1
Dihydrostresptomycin_2
Glucose 13C_2
Colchicine
ns
0.0078
0.0391
0.0234
Peak intensity
Trypsin
Organic Solvent

(B)

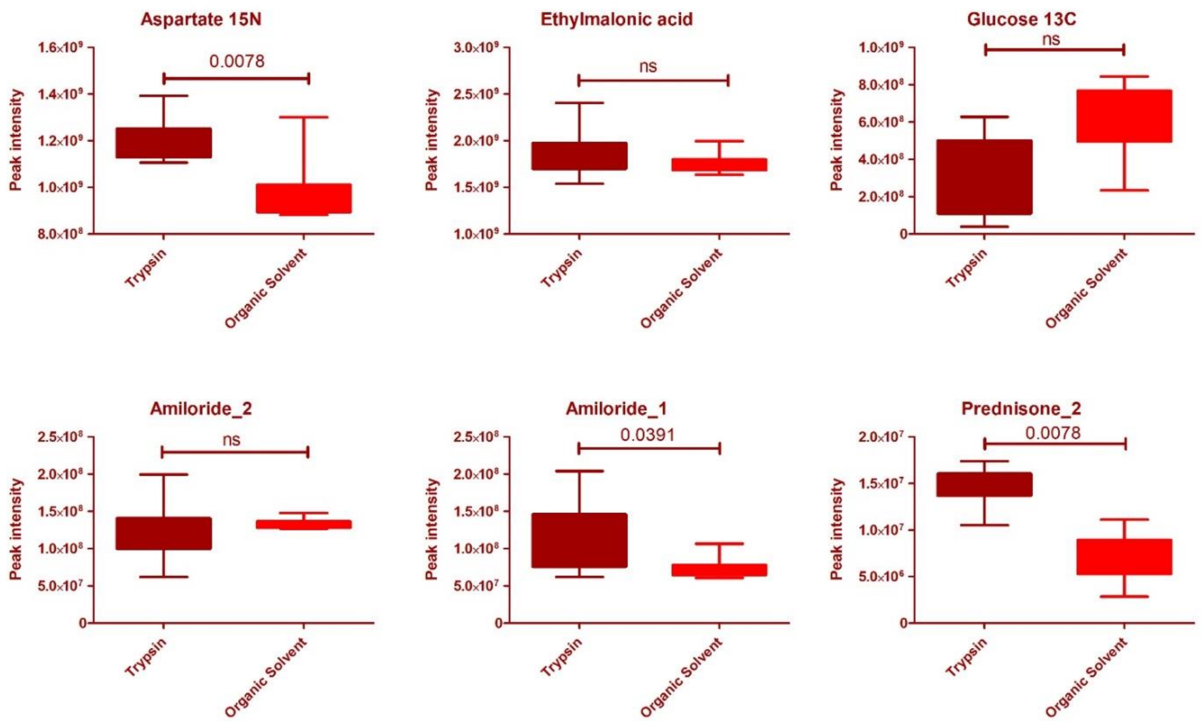

Figure S4: Impact of the two sample preparation protocols on the concentrations of external standards during the LC-HRMS analysis in C18(+) (A) and HILIC(-) (B) conditions: (i) use of trypsin to collect the cells followed by a methanolic extraction (trypsin) or (ii) use of the quenching method and simultaneous extraction of the intracellular metabolites by cold methanol (organic solvent).

	R2X(cum)	R2Y(cum)	Q2(cum)	RMSEE	pre	ort	pR2Y	pQ2
1	0.467	0.649	0.23	0.32	2	0	0.42	0.11

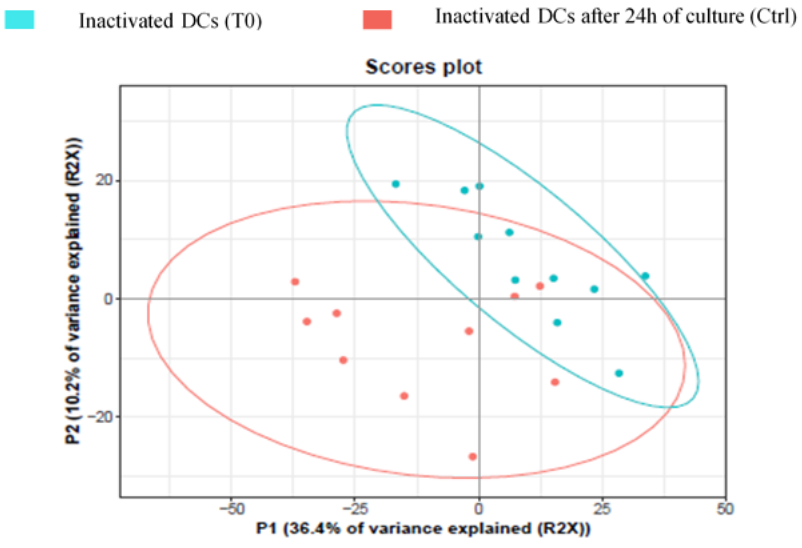

Figure S5: Multivariate supervised statistical analysis was performed on samples from DCs directly after differentiation under GM-CSF and IL-4 during 5 days (T0, blue) and after differentiation +24h of

cell culture in more (Ctrl, red). PLS-DA score plot (R2 = 0.649 and Q2 = 0.23); Data from C18 (+) analyses, 10log transformed.

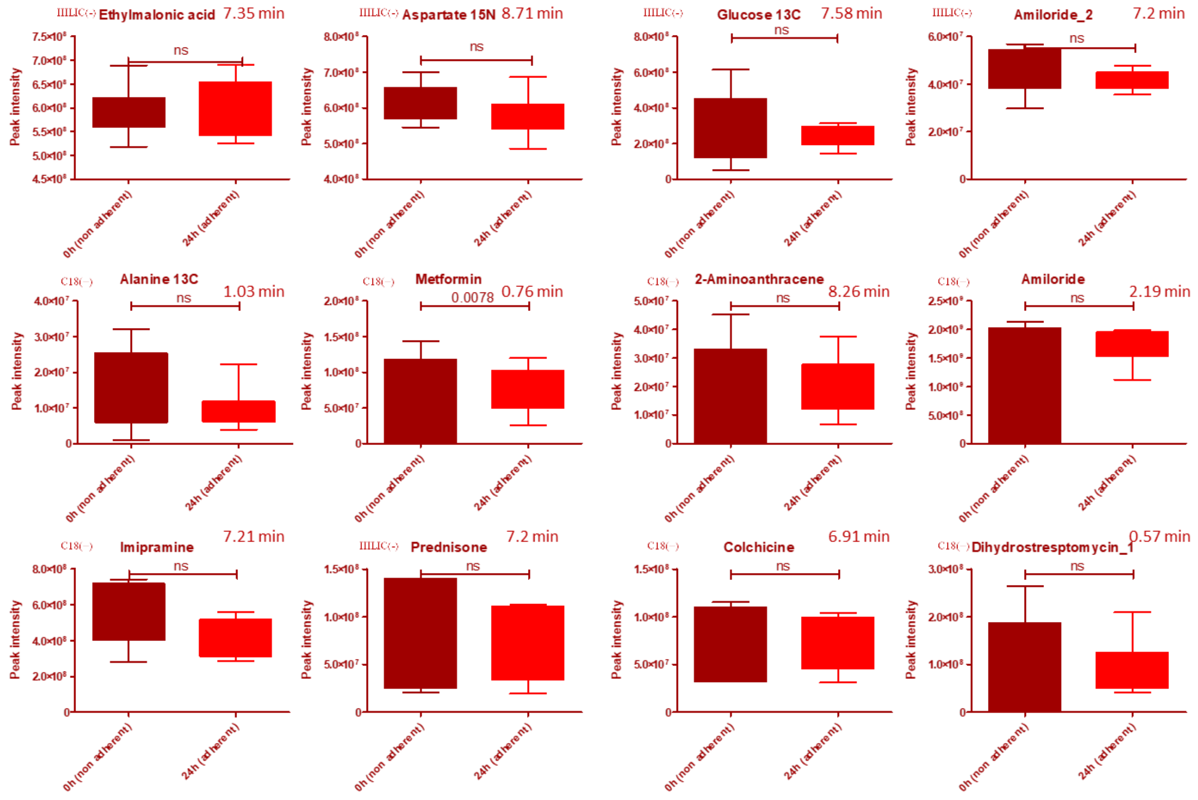

Figure S6: Impact of the cellular state [adherent (0 h), non-adherent (24 h)] on concentrations of external standards during the analysis of the samples analyzed under HILIC (-) and C18 (+) conditions.

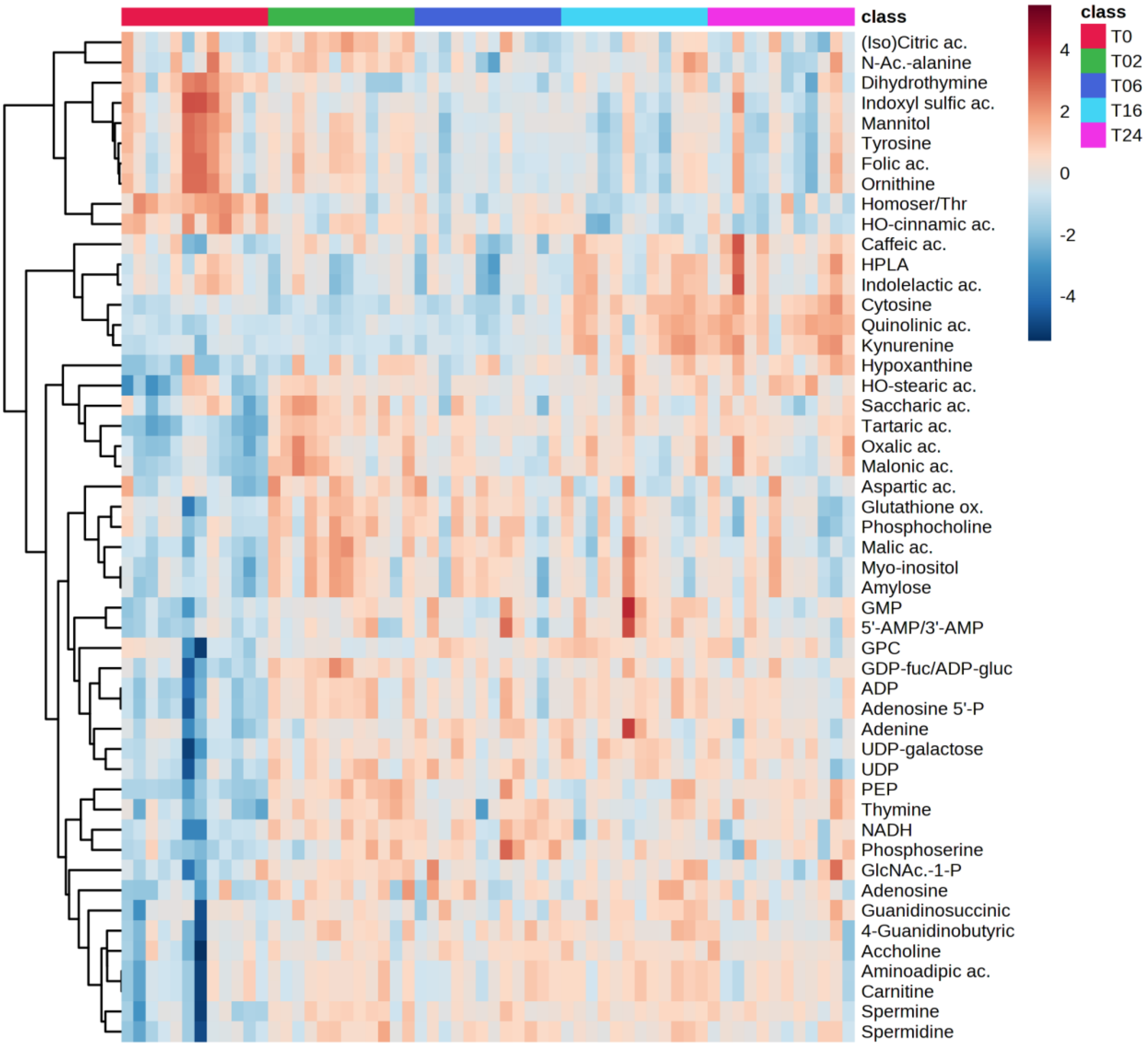

Figure S7: Heatmap obtained from unsupervised analysis of the 12 cell samples collected during a 24h kinetics. Presentation of the 62 most significant metabolites from the t-test/ANOVA analysis (Distance measure: Pearson, Clustering method: Mean). Three large clusters can be distinguished, which correspond to (i) decreased metabolite concentrations within the first two hours, (ii) increased metabolite concentrations after the first 6 hours, and at last increased metabolite concentrations after 2 hours.
